# Changing Face of Radical Prostatectomy: A Nationwide Registry Study of Case-Mix, Surgical Evolution, and Outcomes

**DOI:** 10.3390/cancers18121942

**Published:** 2026-06-14

**Authors:** Martin Schaub, Nicolas Arnold, Raphael Röthlisberger, Swiss Urology Registry Collaborative Group, Daniel Phat Nguyen, Dominik Abt, Agostino Mattei, Räto Strebel, George Thalmann, Laila Schneidewind, Beat Roth, Nicola Giudici

**Affiliations:** 1 Department of Urology, University Hospital of Bern, 3010 Bern, Switzerland; martin.schaub@insel.ch (M.S.);; 2 Department of Urology, Réseau Hospitalier Neuchâtelois, 2000 Neuchâtel, Switzerland; 3 Department of Urology, Spitalzentrum Biel, Centre Hospitalier Bienne, 2501 Biel, Switzerland; 4 Department of Urology, University of Lucerne, Luzerner Kantonsspital, 6000 Lucerne, Switzerland; 5 Department of Urology, Cantonal Hospital of Graubünden, 7000 Chur, Switzerland

**Keywords:** radical prostatectomy, prostate cancer, case-mix, surgical outcomes, MRI-targeted biopsy, PSA persistence

## Abstract

**Background/Objectives:** We aimed to evaluate temporal changes in patient selection, surgical practice, and postoperative outcomes of radical prostatectomy in Switzerland (2020–2025) using a national registry. **Methods:** Data from 7687 patients undergoing radical prostatectomy were extracted from a prospective national registry (January 2020–March 2025). Preoperative, operative, and postoperative variables were compared descriptively using the Mann–Whitney U test for continuous variables and the chi-square test for categorical variables. Temporal trends were assessed using locally estimated scatterplot smoothing. Multivariable logistic regression analyses were performed for positive surgical margins and PSA persistence. **Results:** Baseline patient characteristics remained stable over time. The use of MRI-targeted biopsy increased from 46% to 84% (*p* < 0.001). The proportion of low-risk diseases being treated decreased from 7.1% to 4.0% (*p* = 0.040). Minimally invasive surgery increased from 81% to 95% (*p* < 0.001); pelvic lymph node dissection declined from 83% to 63% (*p* < 0.001). Operative time (205 vs. 185 min, *p* = 0.002) and blood loss (300 vs. 200 mL, *p* < 0.001) decreased over time. Lymph node invasion decreased from 11% to 6.0% (*p* = 0.001), positive surgical margins from 27% to 22% (*p* = 0.044), and prostate-specific antigen persistence from 18.5% to 11.3% (*p* = 0.001). Severe postoperative complications decreased from 2.5% to 0.8% (*p* = 0.014). After multivariable adjustment, surgery performed in 2024 remained independently associated with lower rates of positive surgical margins (OR 0.73, 95% CI 0.57–0.93) and PSA persistence (OR 0.26, 95% CI 0.19–0.37) compared with 2020. **Conclusions:** Using the Swiss national registry, we demonstrate that radical prostatectomy has evolved toward more risk-adapted patient selection, near-universal minimally invasive surgery, and more selective pelvic lymph node dissection. Despite a shift away from low-risk disease, perioperative and early oncological outcomes improved, likely reflecting a combination of evolving diagnostic pathways, increasing surgical experience, and broader changes in perioperative management.

## Simple Summary

This nationwide study evaluates how radical prostatectomy in Switzerland has changed over time. Using data from a large national registry, we show that surgery is increasingly performed in higher-risk patients, with more frequent use of minimally invasive techniques and improved outcomes. These findings highlight how advances in diagnostics and surgical experience are reshaping prostate cancer care in real-world practice.

## 1. Introduction

The management of localized prostate cancer is guided by individual risk stratification. For patients with low-risk prostate cancer (PCa), international guidelines recommend active surveillance to avoid overtreatment and preserve quality of life. For intermediate- and high-risk localized disease, radical prostatectomy is a recommended curative treatment option, alongside radiation therapy [1,2]. Over the past two decades, robot-assisted radical prostatectomy has largely replaced open surgery, as its minimally invasive approach is associated with reduced perioperative morbidity [3,4]. Adoption has expanded rapidly worldwide, particularly in Western countries [5,6].

In parallel, national procedural volumes have increased over time. Based on data from the Swiss national registry, the annual number of radical prostatectomies rose from 3288 cases to 3679 cases between 2021 and 2023 [7]. This upward trend likely reflects a combination of demographic changes, including an aging population, increased detection of PCa through more widespread use of diagnostic strategies, and the highly medicalized Swiss healthcare system with broad access to specialized care. International data suggest that increasing radical prostatectomy volumes and shifting case-mix are not unique to Switzerland. In a European multicenter analysis (four referral centers, 2000–2015), absolute radical prostatectomy numbers rose substantially (401 to 2504) [8]. Similarly, a US population-based study (2000–2009) reported rising prostatectomy volumes (8115 to 10,241) with increasing centralization at very high-volume centers, likely driven in part by the diffusion of robotic surgery [9].

Overall, radical prostatectomy remains a cornerstone treatment for localized PCa and continues to evolve, with particularly rapid advances over the past decade. Alongside the technical innovations, perioperative diagnostic pathways have also changed substantially. In this nationwide registry-based study, we used data from 29 Swiss centers to examine how these developments have influenced real-world practice between 2020 and 2025. Specifically, we aimed to describe whether advances in surgical technique and perioperative diagnostics have altered patient selection, procedural characteristics, and postoperative outcomes, thereby characterizing evolving case-mix and treatment patterns over time.

## 2. Materials and Methods

### 2.1. Data Source

This study uses data from the national, prospective registry of SWISS UROLOGY, a quality-monitoring initiative launched in 2020, with the overarching aim of assessing outcomes across Switzerland. Participation in the registry increased over time. In 2020, 473 radical prostatectomies were recorded, corresponding to 14% of the 3284 procedures performed nationwide. This proportion increased to 41% in 2021 and 50% in 2022 and remained stable at around 50% in 2023 and 2024, reflecting a progressive expansion and subsequent stabilization of registry coverage over the study period. Since 1 July 2025, participation has been mandatory for certified urological training centers, increasing national coverage to ~90%. The structure, data quality, and validity of the registries have been described previously, including analyses demonstrating their applicability for outcome research [10,11]. Data are entered into a standardized electronic platform (Adjumed, Zurich, Switzerland) using a prospective data capture process. All data are pseudonymized before central aggregation. The registry systematically documents patient-, tumor-, treatment-, and outcome-related variables, with follow-up at 3, 12, and 24 months to capture disease course, complications, adjuvant management, and survival. Data quality is ensured through periodic audits coordinated by SWISS UROLOGY.

The study was approved by the Cantonal Ethics Committee of Zurich (BASEC-Nr. Req-2017–00283).

### 2.2. Patient Population

A total of 7687 patients who underwent radical prostatectomy for PCa between January 2020 and March 2025 were included. Procedures were performed using an open, laparoscopic, or robot-assisted approach, according to institutional standards and surgeon preferences. Missing data were low for most baseline, operative, and pathological variables as reported in Table 1. However, postoperative PSA follow-up data were substantially more incomplete in the most contemporary cohort because follow-up collection is still ongoing within the registry. Therefore, analyses involving PSA persistence were restricted to patients with available follow-up data.

### 2.3. Study Outcomes

Outcomes of interest were grouped into preoperative, operative, and postoperative characteristics. Preoperative outcomes included age, ASA (American Society of Anesthesiologists) score, PSA at diagnosis, clinical T stage, biopsy ISUP grade group 1, use of MRI-targeted biopsy, and D’Amico risk classification (low, intermediate, high risk). Operative outcomes included surgical approach (laparoscopic/robot-assisted vs. open), performance of pelvic lymph node dissection, number of lymph nodes removed (if lymph node dissection was performed), operative time, and estimated blood loss. Postoperative outcomes comprised lymph node invasion, positive surgical margin status, PSA persistence (≥0.1 ng/mL at 3 months after surgery), any 30-day complication and 30-day severe complications (Clavien–Dindo ≥ 3).

To further evaluate temporal changes in postoperative outcomes while accounting for differences in patient and disease characteristics over time, multivariable logistic regression analyses were performed for positive surgical margins and PSA persistence. To ensure sufficient follow-up for PSA persistence, adjusted analyses compared patients undergoing surgery in 2020 versus 2024. These outcomes were selected because they were clinically relevant and supported by sufficient event numbers for adjusted modeling. Lymph node invasion was not included because preoperative staging modalities were unavailable and likely changed substantially over time, particularly with the increasing adoption of PSMA PET/CT, which likely had a major impact on staging quality and nodal disease detection. Severe postoperative complications were too infrequent for robust multivariable modeling.

### 2.4. Statistical Analysis

Continuous variables were summarized as medians with interquartile ranges and categorical variables as frequencies and percentages. Continuous and categorical variables were compared descriptively between 2020 (January to December) and the most recent period (March 2024 to March 2025). Because PSA persistence was the only outcome requiring longer postoperative follow-up, it was analyzed separately using two complete calendar years with adequate follow-up availability. Specifically, PSA persistence was compared between patients undergoing radical prostatectomy in 2020 and those treated in 2024. PSA persistence was defined as PSA ≥ 0.1 ng/mL at first postoperative follow-up. Missing follow-up data were reported separately, and percentages were calculated among patients with available PSA follow-up. Differences between groups were assessed using the Mann–Whitney U test for continuous variables and the chi-square test for categorical variables. To visualize temporal trends, locally estimated scatterplot smoothing was applied to continuous calendar time for both continuous and binary outcomes, with the smoothed curves representing locally averaged estimates and 95% confidence intervals.

Multivariable models were adjusted for age, body mass index, D’Amico risk classification, hospital type (university vs. non-university hospitals), ASA score (I–II vs. ≥III), and surgical technique (minimally invasive vs. open/conversion). The year of surgery (2020 vs. 2024) was included as the primary exposure variable. Odds ratios (ORs) with 95% confidence intervals (CIs) were reported.

A complete-case approach was used for multivariable analyses. For PSA persistence, analyses were restricted to patients with available postoperative PSA follow-up data. Overall, PSA follow-up data were missing in 72 of 473 patients (15.2%) in 2020 and in 1098 of 1781 patients (61.7%) in 2024, and these patients were excluded from the adjusted PSA persistence analysis. Missing data among the remaining model covariates were limited, with 87 of 2254 patients (3.9%) having at least one missing covariate value and therefore excluded from adjusted analyses.

All analyses were performed in R version 4.4.1 (R Foundation for Statistical Computing, Vienna, Austria).

## 3. Results

### 3.1. Patient Characteristics

A total of 7687 patients who underwent radical prostatectomy between January 2020 and March 2025 were included. For comparative analyses, the cohort was stratified into two time-based subgroups: patients treated in 2020 (full year; *n* = 473) and those treated in late 2024 to early 2025 (*n* = 1086). Baseline patient, tumor, and surgical characteristics of the overall cohort are summarized in Table 1 and Table 2.

### 3.2. Case-Mix Evolution: Preoperative Characteristics (Figure 1)

Patient demographics remained largely stable, with no significant differences in age or comorbidity burden as reflected by comparable ASA score distributions. In contrast, several clinically and statistically significant shifts were observed in diagnostic and oncological characteristics. Median PSA at diagnosis decreased modestly (8 vs. 7 ng/mL), while the proportion of locally advanced cases (clinical T stage ≥3) treated remained comparable between periods (*p* = 0.2). The use of MRI-targeted biopsy increased markedly from 46% to 84% (*p* < 0.001). Biopsy ISUP grade group distribution shifted significantly between periods, with a substantial reduction in the proportion of surgically treated ISUP grade group 1 disease (16% vs. 9.5%, *p* < 0.001). These shifts were mirrored in the D’Amico risk stratification, with a significant reduction in low-risk disease (7.1% vs. 4.0%, *p* = 0.040).

**Figure 1:**
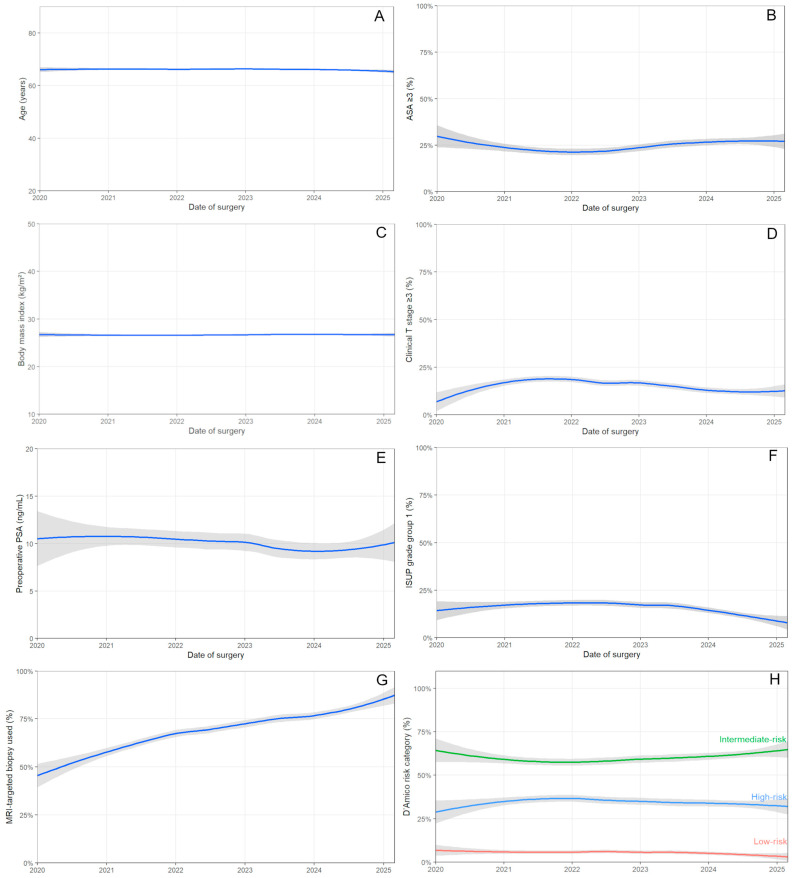
Case-mix evolution–preoperative characteristics. Locally weighted scatterplot smoothing (LOESS) curves are shown with shaded areas representing 95% confidence intervals. The *x*-axis represents the date of surgery. (**A**) Age (years). (**B**) Proportion of patients with ASA score ≥ 3 (%). (**C**) Body mass index (kg/m^2^). (**D**) Proportion of patients with clinical T stage ≥ 3 (%). (**E**) Preoperative prostate-specific antigen (PSA, ng/mL). (**F**) Proportion of biopsy ISUP grade group 1 tumors (%). (**G**) Use of MRI-targeted biopsy (%). (**H**) Distribution of D’Amico risk categories (%), including low-, intermediate-, and high-risk disease. Abbreviations: ASA, American Society of Anesthesiologists; ISUP, International Society of Urological Pathology; PSA, prostate-specific antigen.

### 3.3. Case-Mix Evolution: Operative Characteristics (Figure 2)

Minimally invasive surgery remained the predominant operative approach in both periods but increased significantly over time, from 81% in 2020 to 95% in 2025 (*p* < 0.001). The use of nerve-sparing techniques also increased significantly, from 64% to 75% (*p* < 0.001). In parallel, the rate of pelvic lymph node dissection decreased significantly from 83% to 63% (*p* < 0.001). Among patients undergoing lymph node dissection, the median number of lymph nodes removed declined from 15 to 13 nodes (*p* < 0.001), a difference of limited clinical magnitude. Median operative time decreased significantly from 205 to 185 min (*p* = 0.002), and median estimated blood loss decreased from 300 to 200 mL (*p* < 0.001).

**Figure 2:**
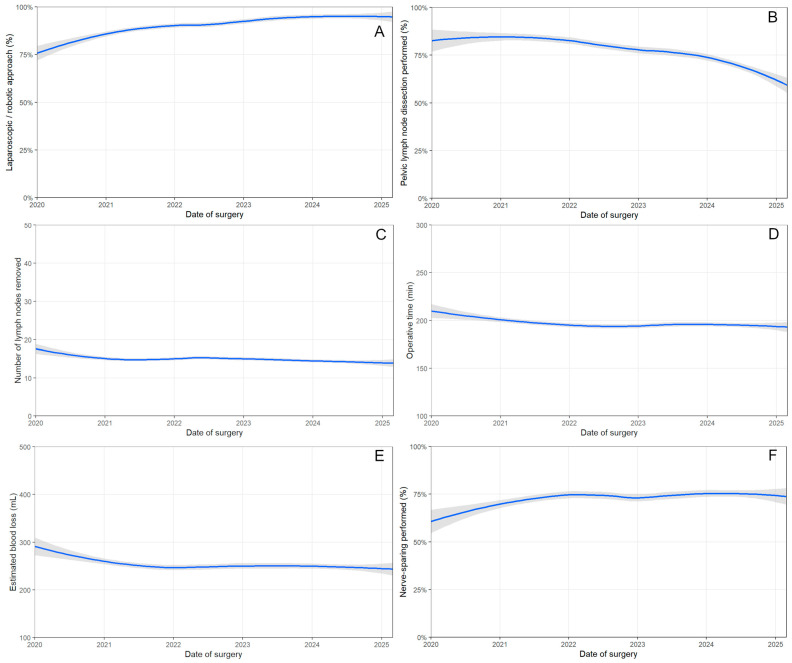
Case-mix evolution–operative characteristics. Locally weighted scatterplot smoothing (LOESS) curves are shown with shaded areas representing 95% confidence intervals. The *x*-axis represents the date of surgery. (**A**) Use of minimally invasive surgery (laparoscopic/robot-assisted approach, %). (**B**) Performance of pelvic lymph node dissection (PLND, %). (**C**) Number of lymph nodes removed (median). (**D**) Operative time (minutes). (**E**) Estimated blood loss (mL). (**F**) Use of nerve-sparing technique (%). Abbreviations: PLND, pelvic lymph node dissection.

### 3.4. Case-Mix Evolution: Postoperative Characteristics (Figure 3)

Postoperatively, the proportion of patients with lymph node invasion decreased significantly from 11% in 2020 to 6.0% in 2025 (*p* = 0.001). Rates of positive surgical margins showed a modest but statistically significant reduction over time, declining from 27% to 22% (*p* = 0.044). PSA persistence decreased from 18.5% in 2020 to 11.3% in 2024 (*p* = 0.001). Overall postoperative morbidity remained stable, with no significant difference in the rate of any postoperative complication between periods. In contrast, severe complications (Clavien–Dindo ≥ 3) decreased significantly from 2.5% to 0.8% (*p* = 0.014), indicating improved perioperative safety despite evolving disease characteristics.

**Figure 3:**
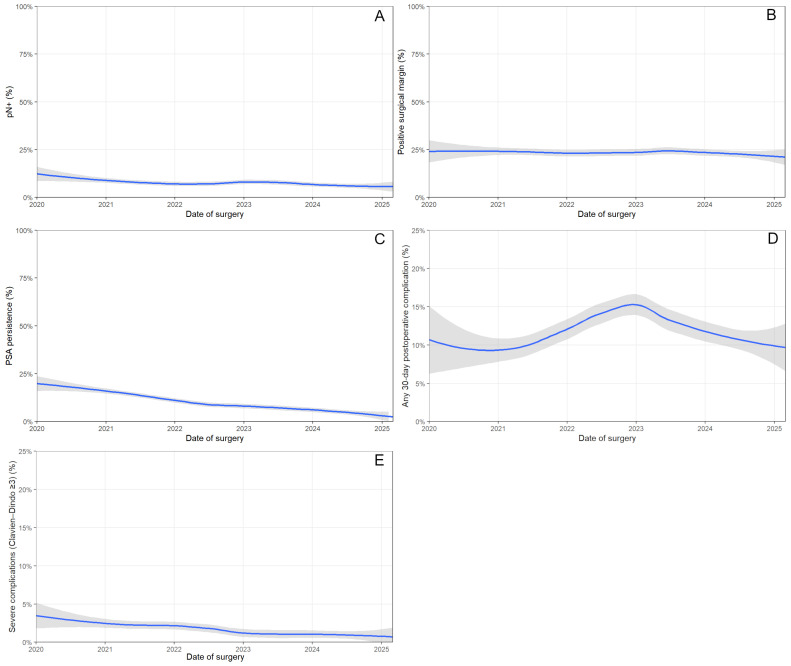
Case-mix evolution–postoperative characteristics. Locally weighted scatterplot smoothing (LOESS) curves are shown with shaded areas representing 95% confidence intervals. The *x*-axis represents the date of surgery. (**A**) Lymph node invasion (pN+, %). (**B**) Positive surgical margin (%). (**C**) Prostate-specific antigen (PSA) persistence (≥0.1 ng/mL at first postoperative follow-up, %). (**D**) Any 30-day postoperative complication (%). (**E**) Any 30-day severe complications (Clavien–Dindo grade ≥ 3, %). Abbreviations: PSA, prostate-specific antigen.

After multivariable adjustment, surgery performed in 2024 remained independently associated with lower rates of positive surgical margins (OR 0.73, 95% CI 0.57–0.93, *p* = 0.011) and PSA persistence (OR 0.26, 95% CI 0.19–0.37, *p* < 0.001) compared with 2020 (Table 3). High-risk D’Amico classification was independently associated with both positive surgical margins and PSA persistence. Hospital type, ASA score, and minimally invasive surgical approach were not significantly associated with positive surgical margins. Complete multivariable analyses are presented in Supplementary Table S2.

## 4. Discussion

In this nationwide registry-based study, overall patient demographics and baseline clinical characteristics remained largely stable over time, whereas marked changes were observed in diagnostic pathways, surgical practice, and postoperative outcomes. Despite a relatively short time frame, we observed a substantial increase in MRI-targeted biopsy and minimally invasive surgery, a reduction in the surgical treatment of low-risk disease, and measurable improvements in operative efficiency and perioperative safety.

Despite the dynamic period studied, patient age and comorbidity burden remained largely unchanged, suggesting that temporal differences in outcomes are unlikely to reflect shifts in baseline surgical fitness. In contrast, we observed meaningful changes in oncological risk distribution and patient selection. The proportion of surgically treated D’Amico low-risk disease decreased significantly from 7.1% to 4.0% (*p* = 0.040), paralleled by a reduction in biopsy ISUP grade group 1 tumors from 16% to 9.5% (*p* < 0.001). These findings likely reflect the growing adoption of active surveillance for low-risk PCa following guideline recommendations [1,2]. This pattern aligns with population-based and registry studies showing a shift in radical prostatectomy toward higher-risk disease, with reduced treatment of low-risk tumors and an increasing proportion of intermediate- and high-risk patients. European multicenter data similarly demonstrate a decline in low-risk cases alongside a substantial rise in high-risk disease [8,12,13]. Concurrently, the marked increase in MRI-targeted biopsy, from fewer than half of patients in 2020 to more than 80% by 2025, underscores the rapid integration of multiparametric MRI into contemporary diagnostic pathways. This shift followed the 2020 EAU guideline update recommending pre-biopsy mpMRI with targeted biopsy when positive [14]. These changes likely also contributed to grade migration and the reduced surgical treatment of favorable-risk disease.

When considering trends in operative features, not surprisingly robotic surgery was increasingly implemented, rising from 81% of procedures in 2020 to 95% in 2025 (*p* < 0.001). In line with previous studies, this shift was accompanied by a significant reduction in intraoperative blood loss (median 300 vs. 200 mL, *p* < 0.001) and shorter operative time (median 205 vs. 185 min, *p* = 0.002) [15]. These improvements likely reflect the progressive maturation of robotic programs, increasing surgeon experience, and ongoing optimization of perioperative workflows.

The role of pelvic lymph node dissection is extensively debated in the literature. In the context of evolving diagnostic tools, such as PSMA PET imaging, real-time intraoperative staging techniques, improved preoperative risk stratification models, and more refined staging approaches, its utilization in clinical practice appears to be progressively reshaped [1,16,17]. In our cohort, we observed a significant decline in the use of pelvic lymph node dissection from 83% in 2020 to 63% in 2025 (*p* < 0.001), accompanied by a modest reduction in nodal yield among patients undergoing dissection over the last five years. Although evolving diagnostic pathways and changing treatment strategies may have contributed to this trend, the underlying reasons cannot be determined from the present registry data, as variables such as PSMA PET imaging and detailed preoperative staging pathways were not systematically captured. Therefore, these findings should be interpreted primarily as descriptive temporal changes in clinical practice.

Within this context, the observed patterns are consistent with the emerging literature that increasingly questions the therapeutic role of lymph node dissection [18,19]. The use of nerve-sparing techniques increased significantly from 64% in 2020 to 75% in 2025 (*p* < 0.001). This likely reflects the increased adoption of MRI and improved imaging interpretation, enabling more precise surgical planning, regardless of the technique used. Notably, this occurred despite a shift toward more advanced disease. The increase was most pronounced between 2020 and 2022, after which it stabilized. Approximately 25% of procedures are still performed without nerve sparing, likely reflecting oncologic considerations. This is consistent with findings from national registry data in Sweden, where the use of nerve-sparing techniques has increased substantially over time but remains lower in patients with higher-risk or more advanced disease, with similar rates observed in our cohort [20].

When considering postoperative features, the rate of PSA persistence decreased significantly from 18.5% to 11.3% in our cohort. This finding is highly relevant, both due to the magnitude of the reduction and because PSA persistence is strongly associated with adverse pathological features and worse oncologic outcomes [21,22]. In the same context, the reduction in positive surgical margins (from 27% to 22%), combined with the decline in PSA persistence over time, suggests improved tumor localization, further experience with robotic platforms, and increasing operator experience, indicating a positive trend in surgical training and education at a national level. Importantly, these findings remained significant after multivariable adjustment for differences in patient and disease characteristics between periods, suggesting that the observed improvements were not solely explained by temporal changes in case-mix. Similar downward trends in margin positivity and pathological stage migration have been reported in other large cohorts over time [23,24]. Whether participation in a national registry, with the opportunity to benchmark outcomes against those of other centers, influenced these results remains an open question.

In line with international trends, we observed a reduction in severe postoperative complications over time. While the overall rate of postoperative complications remained stable (8.5% vs. 11%, *p* = 0.2), the incidence of severe complications (Clavien–Dindo ≥ 3) decreased significantly from 2.5% to 0.8% (*p* = 0.014). This improvement likely reflects advances in surgical technique, perioperative management, and increasing centralization of care. Consistently, large-scale registry data have reported a gradual decline in major complications over time; for example, in the Netherlands, rates decreased from 3.9% to 3.0% following the implementation of minimum volume standards and care centralization [25,26]. Notably, we observed a discrete increase in overall complication rates at the beginning of 2023; however, this was not accompanied by a corresponding rise in severe complications, and no plausible explanatory factor could be identified.

This study has several limitations. First, although based on a prospective national registry, the observational design precludes causal inference and residual confounding cannot be excluded. Multivariable analyses were therefore restricted to selected clinically relevant postoperative outcomes with sufficient event numbers and confirmed the findings of the univariate analyses. While models were adjusted for hospital type, more granular physician- and center-level characteristics, such as surgeon experience, center volume, MRI quality, or radiological expertise, were not available and clustering at the individual center level was not accounted for. Consequently, temporal changes in outcomes may partly reflect unmeasured institutional or provider-related factors, as well as potential selection bias related to evolving registry coverage and differential PSA follow-up completeness over time. Given the substantial proportion of missing PSA follow-up data in the contemporary cohort, the observed temporal improvement in PSA persistence should therefore be interpreted with caution. Furthermore, although regular audit procedures support overall high data quality, reporting or misclassification bias cannot be entirely excluded. Second, due to the multicenter registry design, diagnostic and therapeutic pathways were not standardized and no central radiological or pathological review was performed, introducing variability while reflecting real-world practice. Third, increasing use of advanced imaging, particularly PSMA PET/CT, may have contributed to stage migration; however, these data were not systematically captured, limiting assessment of their impact on the trend of the outcomes. Fourth, long-term oncological endpoints such as biochemical recurrence, cancer-specific survival, and overall survival could not be evaluated owing to the relatively short follow-up inherent to this contemporary cohort and the early timing of this analysis. In addition, functional outcomes such as urinary continence, erectile function, and patient-reported quality-of-life measures were not analyzed. Finally, the generalizability of our findings may be limited to healthcare systems with similar characteristics to Switzerland, which has a highly developed and well-resourced healthcare system with widespread, decentralized access to advanced imaging technologies and robotic surgery across both academic and non-academic centers.

In summary, radical prostatectomy practice in Switzerland between 2020 and 2025 evolved in close alignment with contemporary guideline recommendations. Using high-quality data from a large prospective national registry with minimal missing data, this study captures five-year adoption trends during a period of rapidly evolving diagnostic and therapeutic strategies. Technological and diagnostic advances were accompanied by more selective patient selection and a shift away from low-risk disease, alongside refinement of surgical management, including increased use of minimally invasive and nerve-sparing techniques, reduced pelvic lymph node dissection, and improvements in perioperative safety and early oncological outcomes in a contemporary prostatectomy cohort.

## 5. Conclusions

Using data from the Swiss national registry, we demonstrate that within a highly specialized and modern healthcare system, radical prostatectomy practice between 2020 and 2025 evolved toward more risk-adapted patient selection, near-universal adoption of minimally invasive surgery, and a more selective use of pelvic lymph node dissection. Increased use of MRI-targeted biopsy and fewer low-risk surgeries suggest growing alignment with contemporary guideline recommendations. After multivariable adjustment, surgery performed in 2024 remained independently associated with lower rates of positive surgical margins and PSA persistence compared with 2020. However, given the observational nature of the study, these findings should be interpreted cautiously, as the observed trends likely reflect multiple concurrent changes in diagnostics, surgical practice, and case-mix over time.

## Figures and Tables

**Table 1:** Patient characteristics.

Characteristic	Overall
	N = 7687 ^1^
Age, years	67 (62, 71)
Unknown	8
ASA score	
ASA 1	1321 (17%)
ASA 2	4484 (58%)
ASA ≥ 3	1882 (24%)
Unknown	0
Body mass index, kg/m^2^	26.2 (24.1–29)
Unknown	57
PSA at diagnosis, ng/mL	7 (5, 11)
Unknown	210
Clinical T stage	
cT 1–2	6447 (85%)
cT ≥ 3	1176 (15%)
Unknown	64
Biopsy ISUP grade group	
ISUP 1	1204 (16%)
ISUP 2–3	4858 (64%)
ISUP 4–5	1582 (21%)
Unknown	43
MRI-targeted biopsy	5432 (71%)
Unknown	72
D’Amico risk classification	
Low risk	411 (5.4%)
Intermediate risk	4526 (60%)
High risk	2620 (35%)
Unknown	130
Surgical approach	
Robotic/laparoscopic	6952 (92%)
Open	627 (8.3%)
Unknown	108
Nerve sparing performed	5554 (74%)
Unknown	144
Pelvic lymph node dissection (PLND)	5856 (77%)
Unknown	80
Number of lymph nodes removed (if PLND performed)	11 (3, 17)
Unknown	82
Operative time, min	192 (151, 240)
Unknown	121
Estimated blood loss, mL	200 (100, 300)
Unknown	165
Lymph node invasion (pN1 vs. pN0)	
pN0	6655 (93%)
pN1	526 (7.3%)
Unknown	506
Positive surgical margin	1800 (23%)
Unknown	0
PSA persistence (≥0.1 ng/mL at first follow-up)	690 (9.0%)
Unknown	1107 (14.4%)
Any postoperative complication	906 (12%)
Severe complications (Clavien–Dindo ≥ 3)	118 (1.5%)

^1^ Median (Q1, Q3); n (%).

**Table 2:** Preoperative, operative, and postoperative characteristics by study period (2020 (full year) vs. late 2024/early 2025).

Characteristic	2020 (Full Year) *n* = 473	Late 2024/Early 2025 *n* = 1086	*p* Value
Age, years	67 (62, 71)	66 (61, 71)	0.3
Unknown	0	1	
Body mass index, kg/m^2^	26.2 (24.1, 29.0)	26.2 (24.1, 29.0)	0.8
Unknown	10	47	
ASA score			0.8
ASA 1	105 (22%)	230 (21%)	
ASA 2	238 (50%)	563 (52%)	
ASA ≥ 3	130 (27%)	293 (27%)	
Unknown	0	0	
PSA at diagnosis, ng/mL	8 (5, 12)	7 (5, 10)	0.002
Unknown	13	33	
Clinical T stage			0.2
cT 1–2	426 (90%)	932 (88%)	
cT ≥ 3	46 (9.7%)	128 (12%)	
Unknown	1	26	
Biopsy ISUP grade group			<0.001
ISUP 1	78 (16%)	102 (9.5%)	
ISUP 2–3	311 (66%)	729 (68%)	
ISUP 4–5	84 (18%)	241 (22%)	
Unknown	0	14	
MRI-targeted biopsy	218 (46%)	898 (84%)	<0.001
Unknown	3	17	
D’Amico risk classification			0.040
Low risk	33 (7.1%)	43 (4.0%)	
Intermediate risk	289 (62%)	676 (63%)	
High risk	144 (31%)	346 (32%)	
Unknown	7	21	
Surgical approach			<0.001
Robotic/laparoscopic	372 (81%)	990 (95%)	
Open	87 (19%)	55 (5.3%)	
Unknown	14	41	
Nerve sparing performed	296 (64%)	767 (75%)	<0.001
Unknown	11	59	
Pelvic lymph node dissection (PLND)	382 (83%)	677 (63%)	<0.001
Unknown	12	17	
Number of lymph nodes removed (PLND only)	15 (10, 22)	13 (8, 20)	<0.001
Operative time, min	205 (166, 240)	185 (150, 240)	0.002
Unknown	12	58	
Estimated blood loss, mL	300 (150, 500)	200 (100, 300)	<0.001
Unknown	15	60	
Lymph node invasion (pN1 vs. pN0)			0.001
pN0	388 (89%)	957 (94%)	
pN1	48 (11%)	61 (6.0%)	
Unknown	37	68	
Positive surgical margin	128 (27%)	241 (22%)	0.044
Unknown	0	0	
PSA persistence (≥0.1 ng/mL) ^1^	74 (18.5%)	77 (11.3%)	0.001
Unknown	72 (15.2%)	1098 (61.7%)	
Any postoperative complication	40 (8.5%)	115 (11%)	0.2
Severe complications (Clavien–Dindo ≥ 3)	12 (2.5%)	9 (0.8%)	0.014

^1^ PSA persistence was analyzed separately using complete calendar years (2020, *n* = 473; 2024, *n* = 1781), as this outcome requires postoperative follow-up; therefore, it reflects a different time frame than the other variables presented in this table. Percentages for PSA persistence are calculated among patients with available follow-up data, while missing values are reported separately.

**Table 3:** Multivariable-Adjusted Outcomes for Positive Surgical Margins and PSA Persistence by Year of Surgery.

Variable	PSM Adjusted OR	95% CI	*p*-Value	PSA Persistence Adjusted OR	95% CI	*p*-Value
Year of surgery						
2020 (Ref)	—	—	—	—	—	—
2024	0.73	0.57–0.93	0.011	0.26	0.19–0.37	<0.001

## Data Availability

Requests for access to deidentified data may be considered upon reasonable request to the corresponding author and subject to approval by the Swiss Urology Registry governance and applicable data protection regulations.

## Supplementary Materials

The following supporting information can be downloaded at https://www.mdpi.com/article/10.3390/cancers18121942/s1, Table S1: Collaborators of the Swiss Urology Registry; Table S2: Multivariable logistic regression analyses for positive surgical margins (PSM) and PSA persistence following radical prostatectomy (2020 vs. 2024).
