# Supplementary material for: Changing Face of Radical Prostatectomy: A Nationwide Registry Study of Case-Mix, Surgical Evolution, and Outcomes
Source: Cancers (Basel). 2026 Jun 14;18(12):1942. doi: 10.3390/cancers18121942 (PMC13296577; doi:10.3390/cancers18121942)
Supplement: Supplementary file 1 [file cancers-18-01942-s001.zip › cancers-4336428-supplementary.pdf]

Supplementary

Changing Face of Radical Prostatectomy: A Nationwide Registry Study of Case-Mix, Surgical Evolution, and Outcomes

Supplementary Table S1. Collaborators of the Swiss Urology Registry

Investigator	Center (Switzerland)
Dr. Karim Kellou	Hôpital de Nyon, Nyon, Switzerland
Dr. med. Thomas Sautter	Uroclinic, Wetzikon, Switzerland
Dr. med. Wolfgang Schäfer	Spitalzentrum Oberwallis, Visp, Switzerland
Anja Rieger	Limmattalspital, Schlieren, Switzerland
Dr. med. Simone Brunschweiler	Bellevue Urology, Zürich, Switzerland
Dr. Thomas Tawadros	Hôpital Riviera-Chablais, Rennaz, Switzerland
Dr. med. Thomas Sautter	Uroclinic, Pfäffikon, Switzerland
Dr. Aron Cohen	Urocare AG, Küsnacht, Switzerland
PD Dr. méd. Daniel Nguyen	Hôpital Neuchâtelois, Neuchâtel, Switzerland
Dr. med. Gautier Müllhaupt	Spital Thun (SpitalSTS AG), Thun, Switzerland
Dr. med. Stefan Preusser	Kantonsspital Schaffhausen, Schaffhausen, Switzerland
Prof. Ilaria Lucca	CHUV - Centre Hospitalier Universitaire Vaudois, Lausanne, Switzerland
Prof. Beat Roth	Inselspital, Bern, Switzerland
Dr. Thomas Luginbühl	Spital Uster, Uster, Switzerland
Dr. med. Roland Seiler	Spitalzentrum Biel, Biel, Switzerland
Astrid Bergundthal	Hirslanden Klinik Stephanshorn, St. Gallen, Switzerland
Olivier Ischer & Jérôme Chaptinel	Clinique de la Source, Lausanne, Switzerland
Prof. Massimo Valerio	Hôpitaux Universitaires de Genève (HUG), Genève, Switzerland
PD Dr. med. Räto Strebel	Kantonsspital Graubünden, Chur, Switzerland
PD Dr. Daniel Engeler	Kantonsspital St. Gallen (KSSG), St. Gallen, Switzerland
Prof. Daniel Eberli	Universitätsspital Zürich (USZ), Zürich, Switzerland
PD Dr. med. Tobias Zellweger	St. Claraspital, Basel, Switzerland
Prof. Dr. med. Agostino Mattei	Luzerner Kantonsspital (LUKS), Luzern, Switzerland
Prof. Dr. med. Hubert John	Kantonsspital Winterthur (KSW), Winterthur, Switzerland
Prof. Dr. med. Stephen Wyler	Kantonsspital Aarau, Aarau, Switzerland
Denise Bundi	Uroviva, Bülach, Switzerland
Julien Schwartz	Hirslanden Clinique Cecil, Lausanne, Switzerland
Dr. med. Stephan Bauer	Hirslanden Zentrum für Urologie (ZfU), Zürich, Switzerland

Supplementary Table S2. Multivariable logistic regression analyses for positive surgical margins (PSM) and PSA persistence following radical prostatectomy (2020 vs. 2024). Odds ratios (ORs), 95% confidence intervals (CIs), and p-values are shown.

Variable	PSM Adjusted OR	95% CI	p-value	PSA persistence Adjusted OR	95% CI	p-value
Year of surgery						
2020 (Ref)	—	—	—	—	—	—
2024	0.73	0.57 – 0.93	0.011	0.26	0.19 – 0.37	<0.001
Age	1.02	1.01 – 1.04	0.008	1.00	0.97 – 1.02	0.91
Body mass index (kg/m^2)	1.03	1.01 – 1.06	0.012	0.98	0.93 – 1.02	0.32
ASA score						
I–II (Ref)	—	—	—	—	—	—
≥III	0.97	0.77 – 1.22	0.78	1.12	0.76 – 1.65	0.56
D’Amico risk group						
Low-risk (Ref)	—	—	—	—	—	—
Intermediate-risk	1.33	0.81 – 2.21	0.29	2.06	0.73 – 6.05	0.24
High-risk	2.10	1.26 – 3.53	0.007	7.43	2.65 – 22.1	<0.001
Hospital type						
University hospital (Ref)	—	—	—	—	—	—
Non-university hospital	1.14	0.83 – 1.57	0.43	0.52	0.33 – 0.81	0.006
Surgical technique						
Minimally invasive (Ref)	—	—	—	—	—	—
Open/conversion	1.06	0.75 – 1.51	0.74	2.21	1.41 – 3.49	<0.001
